# Dietary Plant-Derived Phenolic Acids and Phenolamides as Natural Preservatives: Antibacterial, Antioxidant and Food Preservation Applications

**DOI:** 10.3390/foods15122100

**Published:** 2026-06-11

**Authors:** Zhoujing Li, Xin Li, Erzheng Su, Jiasheng Wu, Fangwei Yang

**Affiliations:** 1 State Key Laboratory for Development and Utilization of Forest Food Resources, Nanjing Forestry University, Nanjing 210037, China; 2311510112@njfu.edu.cn (Z.L.); lixin123@njfu.edu.cn (X.L.); ezhsu@njfu.edu.cn (E.S.); 2 College of Light Industry and Food Engineering, Nanjing Forestry University, Nanjing 210037, China; 3 State Key Laboratory for Development and Utilization of Forest Food Resources, Zhejiang A&F University, Hangzhou 311300, China; wujs@zafu.edu.cn; 4 Food Laboratory of Zhongyuan, Luohe 462300, China; 5 Key Laboratory of Food Safety Rapid Detection Technology and Product Evaluation Center for Jiangsu Province Market Regulation, Suzhou Institute for Food Control, Suzhou 215000, China

**Keywords:** phenolic acids, phenolamides, antibacterial, antioxidant, food preservation, food application

## Abstract

Food spoilage from microbial contamination and oxidation drives the search for natural preservatives. Phenolic acids (PAs) and phenolamides are plant-sourced metabolites with broad-spectrum antimicrobial and antioxidant activities. This review comprehensively examines their sources, classification, structure–activity relationships, and multi-target mechanisms. PA antimicrobial action involves membrane disruption, intracellular acidification, and oxygen species generation, while antioxidant effects rely on hydrogen donation and metal chelation. For phenolamides, antimicrobial evidence is largely indirect, based on computational docking and one non-food nucleotide biosynthesis study, and direct validation of these mechanisms in food matrices against common foodborne pathogens is lacking. Delivery strategies (direct incorporation, encapsulation, edible coatings, active packaging) are critically evaluated, with emphasis on PA-grafted chitosan systems. Applications of PAs in fruits, vegetables, meat, aquatic products, and lipid-rich emulsions are summarized. Phenolamide applications are limited by low natural abundance, high purification costs, poor aqueous solubility, and a historical bias toward pharmacology. Safety assessments confirm favorable profiles for many PAs and select phenolamides, though chronic toxicity data for phenolamides remain limited. This review provides a theoretical framework for leveraging PAs and emerging phenolamides as natural preservatives and identifies critical knowledge gaps requiring future investigation.

## 1. Introduction

Food spoilage remains a persistent challenge throughout the global supply chain, encompassing storage, transportation, and retail stages. It is primarily driven by two synergistic processes, namely microbial contamination by foodborne pathogens (e.g., *Staphylococcus aureus*, *Escherichia coli*, and *Listeria monocytogenes*) and oxidative deterioration of endogenous components such as lipids and proteins [1,2,3]. Among these, microbial contamination by spoilage microorganisms and pathogenic bacteria is particularly critical due to its ubiquity and the severe health risks involved [2,3]. These microorganisms are ubiquitous in natural environments, including soil and water, and can colonize food matrices at multiple entry points along the supply chain, posing a continuous threat to food safety [3]. Consequently, high-moisture perishable commodities, such as ready-to-eat products, meat, fish, dairy, fruits, and vegetables, are frequently contaminated by spoilage microorganisms and foodborne pathogens [4].

In tandem with microbial activity, oxidative deterioration is another major driver of food quality loss. Lipid oxidation is a complex radical chain reaction initiated upon exposure of fats and oils to oxygen, thermal stress, or light, ultimately leading to rancidity, nutrient depletion, and the formation of potentially toxic metabolites [5]. Protein oxidation, while often under-researched compared to lipids, is equally important. It occurs when muscle proteins interact with reactive oxygen species (ROS), such as superoxide anions and peroxyl radicals, or non-radical species such as hydrogen peroxide (H_2_O_2_). These reactions are further catalyzed by external stressors (e.g., light and irradiation) and chemical pro-oxidants, including free radicals, transition metal ions (e.g., Fe^2+^), reducing sugars, and pro-oxidative hemoproteins. The effects of protein oxidation include adverse sensory shifts, off-flavors, sourness, unpleasant odors, and discoloration that severely compromise freshness and consumer acceptability. Moreover, oxidative modifications may alter protein conformation and digestibility, thereby compromising nutritional quality and potentially generating toxic secondary oxidation products [6].

The convergence of microbial contamination and oxidative deterioration results in substantial losses in nutritional and sensory integrity, contributing significantly to global food waste, resource depletion, and environmental burdens [7]. Consequently, developing effective and safe preservation strategies is essential for ensuring food safety and mitigating economic losses.

Currently, the food industry relies heavily on synthetic antioxidants and preservatives; however, emerging evidence suggests that certain synthetic additives may induce adverse health effects, including hepatotoxicity, leading to heightened consumer skepticism [8]. This transition has accelerated the search for natural, “clean-label” alternatives. Bioactive compounds derived from edible plants have emerged as promising candidates due to their favorable safety profiles and multifunctional properties. Notably, phenolic acids (PAs) and phenolamides, ubiquitous in dietary plants, herbs, and spices, stand out as key secondary metabolites possessing broad-spectrum antimicrobial and potent antioxidant activities. These attributes render them viable natural substitutes for conventional synthetic additives [9]. Accordingly, this review comprehensively examines the antibacterial and antioxidant mechanisms of dietary plant-derived PAs and phenolamides, evaluates recent advancements in their application, and provides theoretical frameworks for the development of effective natural food preservation systems.

Building on the comprehensive summaries of PA bioactivities available in the recent literature [2,3,10,11], this review uses the well-established knowledge on PAs as a lens to critically examine phenolamides. We focus primarily on high-moisture, non-fermented perishable commodities (fresh produce, meat, seafood, and lipid-rich emulsions). Fermented foods, dairy products, and clear beverages are not covered herein; readers are directed to specialized reviews for those applications. A comprehensive literature search was conducted using the Web of Science, PubMed, and Scopus databases (up to April 2026). Search terms included “phenolic acid”, “phenolamide”, “hydroxycinnamic acid amide”, “food preservation”, “antimicrobial”, and “antioxidant”, combined using Boolean operators. As this is a narrative review, no formal systematic review protocol or predefined inclusion/exclusion criteria were applied. Instead, representative peer-reviewed studies were selected to provide a critical overview of the mechanisms, advances, and unresolved questions in the field.

The principal contribution of this review is the use of PAs as a consolidated framework to critically evaluate the emerging potential and current limitations of phenolamides. By juxtaposing these two classes, we identify transferable methodologies and pinpoint knowledge gaps obscured by the historical pharmacological bias in phenolamide research.

## 2. Types, Sources, and Representative Compounds of PAs and Phenolamides

PAs and phenolamides, particularly hydroxycinnamic acid amides (HCAAs), constitute two important classes of bioactive metabolites widely distributed across the plant kingdom. Their chemical diversity, dietary sources, and food-relevant bioactivities are summarized in Table 1 and Table 2, respectively, with full pharmacological profiles provided in Table S1. A systematic understanding of their classifications, chemical structures, and sources is a prerequisite for optimizing their application in food systems.

### 2.1. Classification, Sources, and Biosynthetic Pathways of PAs

**Figure 1:**
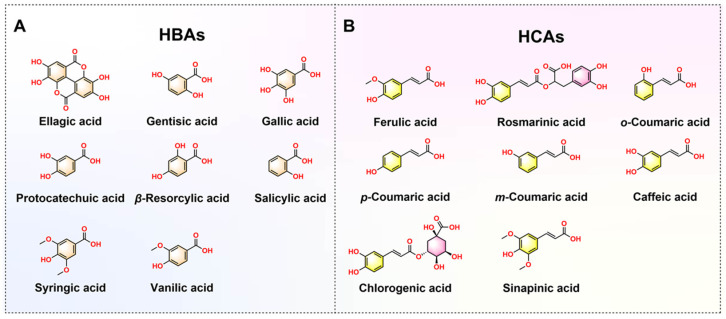
Classification and structural diversity of phenolic acids into hydroxybenzoic acids (HBAs, (**A**)) and hydroxycinnamic acids (HCAs, (**B**)) [3]. Color legend: Orange rings indicate the aromatic ring of hydroxybenzoic acids (HBAs); yellow rings indicate the aromatic ring of hydroxycinnamic acids (HCAs); pink rings indicate other cyclic moieties within HCAs (including additional benzene rings). Red lettering indicates oxygen-containing groups.

As summarized in Table 1, PAs are plant-derived secondary metabolites characterized by an aromatic ring bearing at least one hydroxyl substituent and one carboxyl group [2,34]. As typical products of phenylpropanoid metabolism, they are divided into two major subclasses based on their carbon scaffolds: hydroxybenzoic acids (HBAs, C6–C1 backbone), derived from benzoic acid, and hydroxycinnamic acids (HCAs, C6–C3 backbone) derived from cinnamic acid [3,35,36]. Prominent HBAs (Figure 1A) include gallic acid (GA), *p*-hydroxybenzoic acid (PHBA), salicylic acid, ellagic acid (EA), gentisic acid, protocatechuic acid (PCA), syringic acid, and vanillic acid. The HCA (Figure 1B) subclass primarily includes *p*-coumaric acid, caffeic acid (CA), ferulic acid (FA), sinapic acid, isoferulic acid, and *p*-hydroxycinnamic acid [37]. Among these, compounds such as FA, CA, *p*-coumaric acid, vanillic acid, PHBA, GA, chlorogenic acid (CGA), and rosmarinic acid (RA) have been extensively studied due to their potent bioactivities [9].

**Figure 2:**
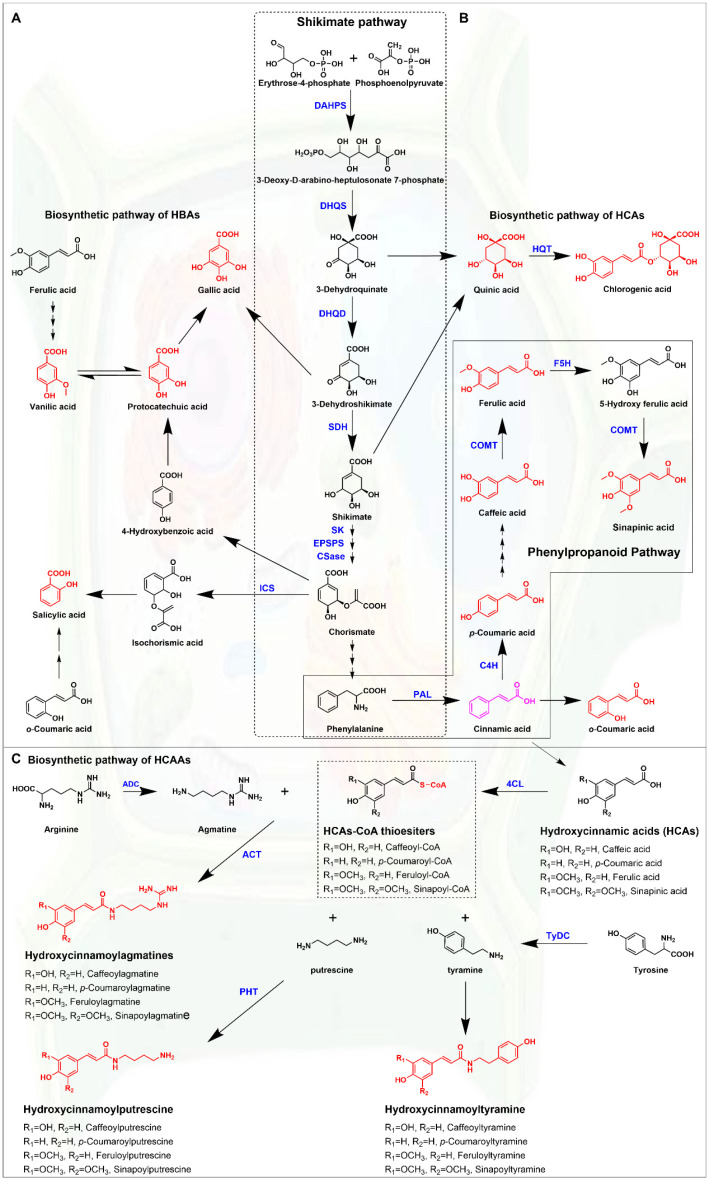
Biosynthetic pathways of hydroxybenzoic acids (HBAs) (**A**), hydroxycinnamic acids (HCAs) (**B**) and phenolamides (**C**) [35,36,38]. Color legend: Red structural formulas represent common PAs/phenolamides; purple structural formula (cinnamic acid) is the key precursor of hydroxycinnamic acids; blue text indicates enzyme abbreviations (see Notes); Black is used for all other structural formulas and text. Notes: DAHPS, 3-deoxy-D-arabinoheptulosonate 7-phosphate synthase; DHQS, 3-dehydroquinate synthase; DHQD, 3-dehydroquinate dehydratase; SDH, shikimate dehydrogenase; SK, shikimate kinase; EPSPS, 5-enolpyruvylshikimate 3-phosphate synthase; CSase, chorismate synthase; HQT, hydroxycinnamoyl-CoA quinate: hydroxycinnamoyl transferase; ICS, isochorismate synthase; PAL, phenylalanine ammonia-lyase; C4H, cinnamate 4-hydroxylase; COMT, cinnamoyl (caffeic) acid 3-O-methyltransferase; F5H, ferulate 5-hydroxylase; ADC, arginine decarboxylase; 4CL, 4-coumarate-CoA: ligase; ACT, agmatine coumaryl transferase; PHT, putrescine hydroxycinnamoyl transferase; TyDC, tyrosine decarboxylase.

The biosynthetic pathways of common HBAs and HCAs are depicted in Figure 2A,B. From a biosynthetic standpoint, both classes originate predominantly from the shikimate pathway, which is initiated by the condensation of glycolysis-derived phosphoenolpyruvate and pentose phosphate pathway-derived erythrose-4-phosphate. This sequence of enzymatic steps yields aromatic amino acids and essential precursors for diverse aromatic metabolites [36,38]. For HBAs, biosynthesis converges at chorismate, the terminal intermediate of the shikimate pathway. From this node, PHBA is generated via chorismate pyruvate-lyase, while salicylic acid is synthesized via isochorismate following isomerization. The biosynthesis of PCA follows dual pathways: the hydroxylation of PHBA or the direct dehydration of 3-dehydroshikimate. GA is derived from the subsequent hydroxylation of protocatechuate, whereas vanillic acid originates either as a lignin degradation product or from the oxidative cleavage of HCAs such as FA [38]. For HCAs, biosynthesis occurs primarily via the phenylpropanoid pathway, starting with phenylalanine. Upon translocation to the cytoplasm, phenylalanine undergoes deamination by phenylalanine ammonia-lyase (PAL) to form cinnamic acid [36,39].

PAs are widely distributed in plant-based matrices such as fruits, cereals, and wine, with substantial concentrations localized in agricultural byproducts, particularly cereal processing residues like wheat bran [34]. Compounds such as FA, *p*-coumaric acid, and PCA are major constituents of lignocellulosic water extracts, offering a sustainable pathway for resource valorization in alignment with circular economy principles [40]. Such valorization of agricultural byproducts not only reduces waste but also provides a sustainable source of natural preservatives and antioxidants.

### 2.2. Representative Dietary PAs and Their Antibacterial and Antioxidant Activities

The classification and biological activities of PAs have been extensively reviewed elsewhere [39]; thus, only representative compounds are highlighted here. While PAs exhibit a broad spectrum of bioactivities (Table S1), this section focuses specifically on their antimicrobial and antioxidant properties, which are most relevant to food preservation.

#### 2.2.1. Benzoic Acid Derivatives

GA, a trihydroxybenzoic acid, is highly water-soluble and exhibits effective antioxidant capacity [41]. Its metabolite, EA, provides supplementary ultraviolet (UV)-blocking effects. PCA has been shown to inhibit major foodborne pathogens, including *Listeria monocytogenes* and *Escherichia coli* [40]. Salicylic acid exhibits moderate antioxidant activity. Benzoic acid derivatives are widely distributed in fruits, nuts, and vegetables [1,3,41].

#### 2.2.2. Hydroxycinnamic Acid Derivatives

These typically exhibit superior antioxidant activity compared to benzoic acids due to their conjugated double bonds and catechol or guaiacyl moieties. CA, possessing two hydroxyl groups, displays higher radical scavenging efficacy than GA both in vitro and in vivo [3,41]. FA acts through dual mechanisms inhibiting pro-oxidative enzymes and enhancing antioxidant enzymes, though its sensitivity to oxidation during processing remains a challenge [3,7]. Sinapic acid (3,5-dimethoxy-4-hydroxycinnamic acid) displays strong anti-inflammatory and anxiolytic effects [41]. CGA (CA esterified with quinic acid) functions as an effective hydrogen donor with additional antidiabetic, neuroprotective, and cardiovascular benefits [3]. Notably, RA exhibits the highest radical scavenging capacity, outperforming butylated hydroxytoluene and α-tocopherol by over threefold in specific assays [1]. Collectively, HCAs exhibit a broad spectrum of bioactivities, including antioxidant, anti-inflammatory, neuroprotective, and cardioprotective effects, that underpin their potential both as functional food ingredients and as natural preservatives [42]. While these in vitro activities are well established, the translation of PAs into effective food preservatives faces practical challenges related to solubility, stability and food matrix interactions; these issues are critically examined in Section 4 and Section 5.

### 2.3. Types, Sources, and Biosynthetic Pathways of Phenolamides

**Figure 3:**
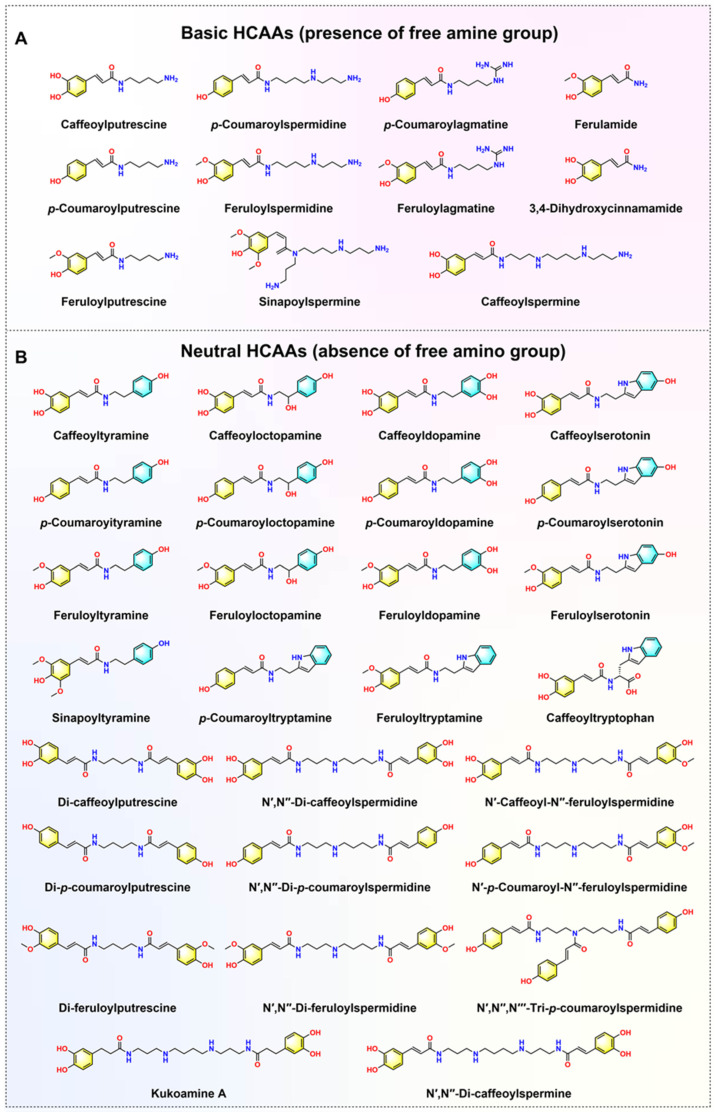
Classification and structural diversity of hydroxycinnamic acid amides (HCAAs) based on amine moiety properties. (**A**) Basic group; (**B**) Neutral group [47]. Color legend: Yellow rings indicate the cinnamic acid-derived benzene rings; blue rings indicate the amine-derived benzene rings. Red lettering indicates oxygen-containing groups; blue lettering indicates amino-containing groups.

**Figure 4:**
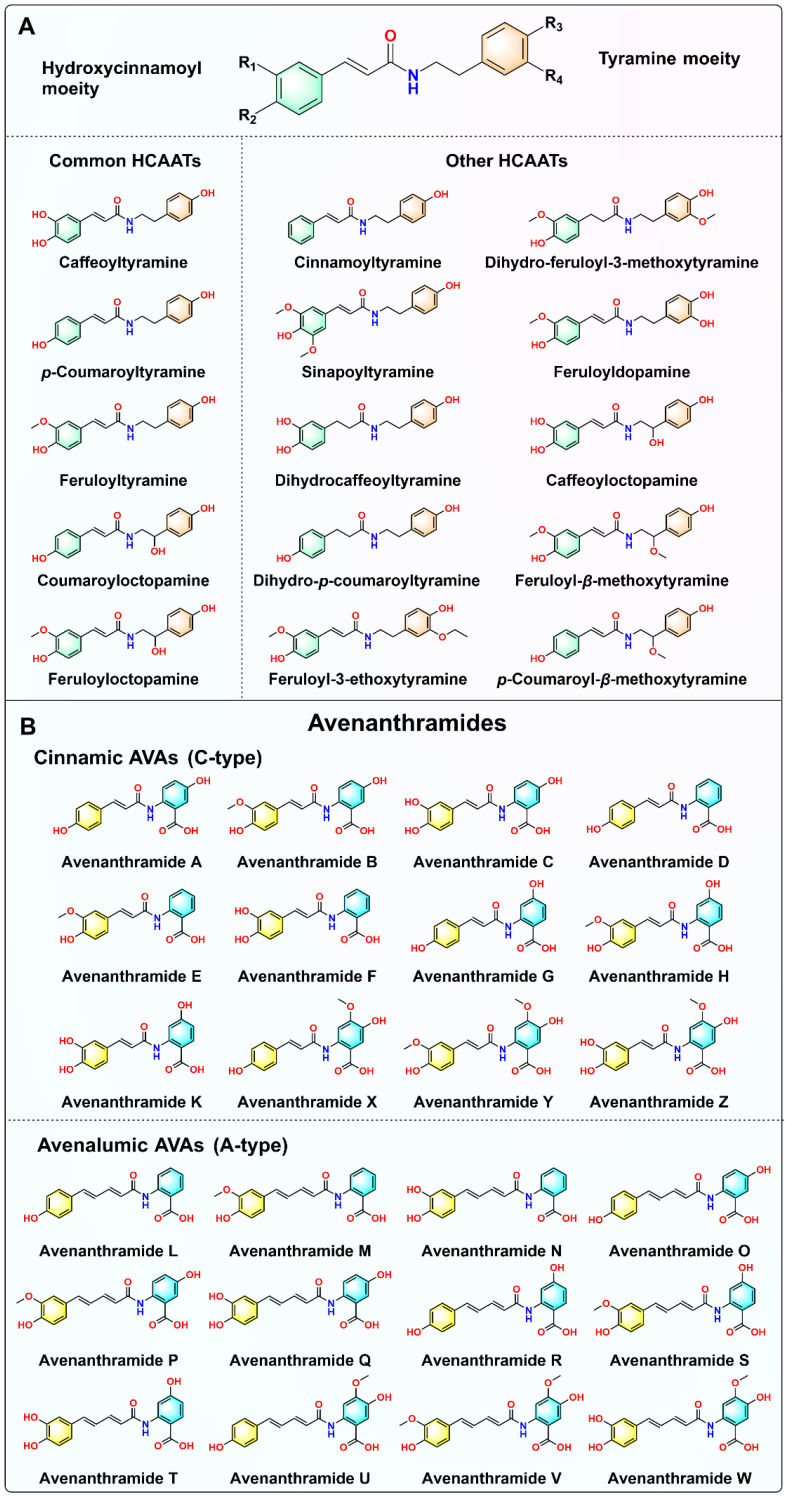
Chemical structures of hydroxycinnamic acid tyramine amides (HCAAT) derivatives (**A**) and avenanthramides (**B**) [44,48]. Color legend for benzene rings: In part A, cinnamic acid-derived benzene rings are green; amine-derived benzene rings are orange. In part B, cinnamic acid-derived benzene rings are yellow; amine-derived benzene rings are blue. Letter colors as in Figure 3.

As illustrated in Table 2 and Figure 3, phenolamides, especially HCAAs, are an important class of plant secondary metabolites. They are synthesized by conjugating HCAs with various amines via amide linkages and often serve as structural components within plant cell walls by crosslinking with cell wall polysaccharides including pectins and extensins [43]. These compounds are integral to plant defense and adaptation and are found in edible plants such as oats, goji berries, tomatoes, and rice [44]. Since their discovery in the 1940s, the structural diversity of HCAAs has been extensively characterized, governed by enzyme-catalyzed condensation between hydroxycinnamoyl-CoA and aliphatic or aromatic amines, a process mediated by specific hydroxycinnamoyl transferases (Figure 2C) [45,46]. Based on the chemical properties of the amine moiety, HCAAs are classified into two groups: the basic group (hydrophilic and ionizable, Figure 3A) and the neutral group (hydrophobic, with lower water solubility, Figure 3B) [47]. Based on the core structure of the conjugated amine, phenolamides can be further categorized into several subclasses, including aromatic monoamine-conjugated phenolamides that are derived from conjugation with tyramine, octopamine, dopamine, etc.; aliphatic diamine/polyamine-conjugated phenolamides that are formed by conjugating with putrescine, spermidine, etc.; and avenanthramides formed with anthranilic acid derivatives [44]. The structural library of plant amine conjugates has expanded far beyond the 38 aromatic monoamine and 36 aliphatic diamine/polyamine conjugates originally documented in edible plants by Wang et al. [44]. Leveraging high-resolution mass spectrometry and multi-omics approaches, numerous new conjugates have subsequently been characterized, shifting the current understanding from mere structural diversity toward their regulatory roles in plant stress responses and immunity [43,44,46]. As shown in Figure 3, nearly all PA moieties that constitute phenolamides are derived from HCAs, including *p*-coumaric acid, CA, FA, and sinapic acid. In contrast, natural phenolamides derived from HBAs are rarely reported. This disparity largely arises from the substrate specificity of the key biosynthetic enzymes, *N*-hydroxycinnamoyl transferases, which preferentially utilize HCAs as acyl donors to form amide bonds with various amines [43,46]. Among the aromatic monoamine conjugates, tyramine-derived hydroxycinnamic acid tyramine amides (HCAATs) (Figure 4) have emerged as one of the most extensively investigated subclasses, owing to their widespread occurrence in various plant-based foods and potent biological activities demonstrated in chemical, cellular, and animal models. Representative HCAATs include *N*-*trans*-feruloyltyramine, *N*-*trans*-coumaroyltyramine, *N*-*trans*-caffeoyltyramine, cinnamoyltyramine, and sinapoyltyramine [47].

### 2.4. Representative Dietary Phenolamides and Their Antibacterial and Antioxidant Activities

As summarized in Table S1, dietary phenolamides exhibit diverse bioactivities, including anticancer, anti-inflammatory, and neuroprotective effects. This section, however, focuses on their antimicrobial and antioxidant activities, which underpin their potential in food preservation. Avenanthramides (Figure 4) represent the signature phenolamides of the *Avena* genus, comprising *ortho*-aminobenzoic acid derivatives conjugated with HCAs like FA or CA [44,48,49]. Li et al. elucidated that A-type avenanthramides scavenge peroxyl radicals via a stepwise proton-loss electron transfer (SPLET) pathway under physiological conditions, exhibiting rate constants significantly superior to those of synthetic antioxidants like BHT and Trolox [21]. Consistently, Wang et al. reported a half-maximal inhibitory concentration (IC_50_) of 7.38 μg/mL for avenanthramide C in 2,2-Diphenyl-1-picrylhydrazyl (DPPH) radical scavenging assays [22]. Although traditionally recognized as fungal inhibitors, their industrial application has historically been hindered by high isolation costs [49,50]. Recent breakthroughs in synthetic biology and microbial cell factories, however, now provide sustainable, large-scale biomanufacturing routes for these high-value metabolites [50].

Among aromatic monoamine conjugates, *N*-*trans*-caffeoyltyramine has emerged as a research focus due to its prevalence in *Lycium* and *Cannabis* [51]. Regarding antioxidant capacity, Boucher et al. demonstrated that *N*-*trans*-caffeoyltyramine exhibits superior DPPH scavenging activity, outperforming its analogs *N*-*trans*-feruloyltyramine, *N*-sinapoyltyramine, and *N*-*trans*-*p*-coumaroyltyramine [52]. Density functional theory (DFT) simulations further revealed that the antioxidant mechanism of *N*-*trans*-caffeoyltyramine is medium-dependent, utilizing hydrogen atom transfer (HAT) in the gas phase but shifts to a SPLET pathway in polar media [53]. Among synthetic derivatives, FA-based amide analogues have demonstrated the highest antioxidant efficacy relative to other cinnamic acid counterparts [54]. The antimicrobial profile of *N*-*trans*-caffeoyltyramine is also noteworthy, characterized by significant antifungal properties and the inhibition of common food spoilage bacteria [55]. Quantitative studies indicate that *N*-*trans*-feruloyltyramine and *N*-*trans*-caffeoyltyramine isolated from *Triclisia sacleuxii* exhibit a Minimum Inhibitory Concentration (MIC) of 7.8 μg/mL against *Staphylococcus aureus*, while *N-trans*-caffeoyltyramine showed an MIC of 15.7 μg/mL. Both values are substantially lower than that of tetracycline (125 μg/mL), although these values were obtained in nutrient media and may not directly predict efficacy in complex food environments. These compounds also exhibit potent activity against *Escherichia coli* and *Pseudomonas aeruginosa* [47]. Furthermore, Dihydro-*N*-caffeoyltyramine, a reduced derivative of N-caffeoyltyramine, has demonstrated efficacy against methicillin-resistant and vancomycin-resistant *Staphylococcus aureus*, with MIC below 50 μg/mL. Additionally, *N*-*trans*-feruloyltyramine from *Lycium* root bark shows significant antifungal activity against *Candida albicans* (5 μg/mL < MIC < 10 μg/mL) [56]. Various cinnamic acid amide analogues from *Allium fistulosum* have also been reported to possess antibacterial potential, although their structural classification as phenolamides requires further verification [36].

*p*-Coumaroylamides are secondary metabolites formed via the amide linkage of *p*-coumaric acid with diverse amines. Ubiquitous across the plant kingdom, from monocotyledonous crops in the Poaceae family (e.g., barley and rice) to various dicotyledonous species, these compounds have attracted interest in natural product research due to their antioxidant, anti-inflammatory, antimicrobial, and anticancer properties [57]. *N*-*trans*-*p*-coumaroyltyramine is among the most extensively characterized representatives. Its antioxidant efficacy has been validated through multiple in vitro models, including ferric reducing antioxidant power, DPPH, H_2_O_2_, and ABTS (2,2′-azino-bis(3-ethylbenzothiazoline-6-sulfonic acid) scavenging assays. Regarding antimicrobial activity, it exhibits broad-spectrum activity, with inhibition zone diameters of 11–18 mm for Gram-positive bacteria (e.g., *Staphylococcus aureus*, *Bacillus subtilis*) and 12–21 mm for Gram-negative pathogens (e.g., *Escherichia coli*, *Shigella*), underscoring its potential as a natural biopreservative [23]. Other notable *p*-coumaroyl derivatives include *p*-coumaroyloctopamine (antioxidant), *p*-coumaroylputrescine (an antioxidant involved in the potato wound response), and *p*-coumaroylagmatine (antifungal) [57].

Significantly, phenolamide dimers have also demonstrated unique antibacterial potential. Recent studies highlight that hydroxycinnamic acid amide dimers, whether chemoenzymatically synthesized or recovered from brewing byproducts like barley roots, effectively inhibit beer-spoilage yeasts. For example, CouAgm-4-O-7′/3-8′-DCouAgm and FerAgm-4-O-7′/3-8′-DFerAgm were active in beer at concentrations of 46–255 μg/mL [58]. Similarly, lyciumamide A, a dimer isolated from *Lycium* fruits, has been identified as a potent antioxidant [33]. Importantly, the antimicrobial data summarized above derive predominantly from standardized in vitro susceptibility assays; direct validation of these activities against foodborne pathogens in real food matrices remains to be established, and extrapolation to food preservation should therefore be made with caution.

## 3. Mechanisms Underlying the Antimicrobial and Antioxidant Activities of PAs and Phenolamides

PAs are important defense metabolites that regulate physiological responses to both biotic and abiotic stressors [34]. Upon dietary ingestion, PAs exhibit an array of health-promoting bioactivities, encompassing antioxidant, antimicrobial, anti-inflammatory, anticancer, and cardioprotective properties [3,37]. In food science, these functionalities enable the inhibition of foodborne pathogens and the attenuation of lipid and protein oxidation, providing a mechanistic basis for natural preservation strategies [2,34].

### 3.1. Antimicrobial Mechanisms of PAs

**Figure 5:**
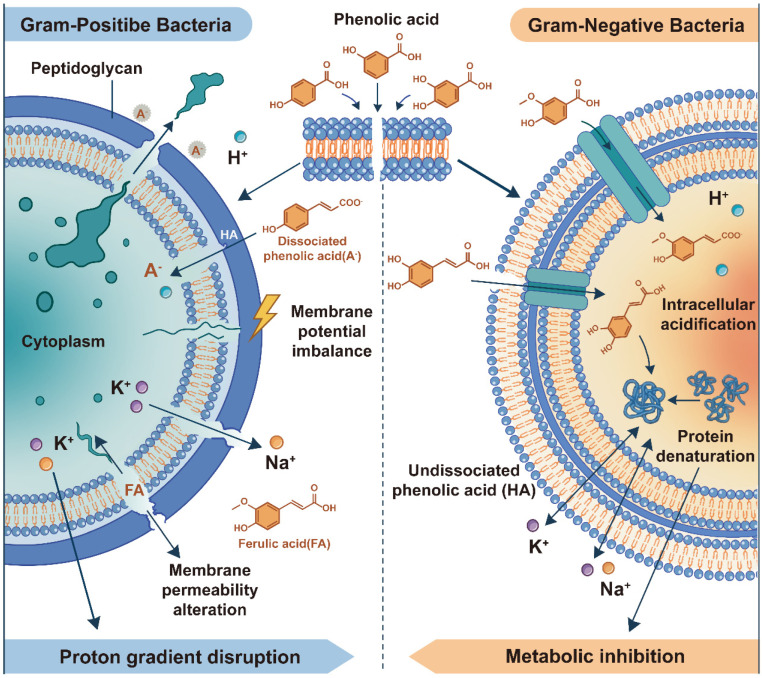
Mechanisms of action of phenolic acids against Gram-positive and Gram-negative bacteria [61]. Notes: PAs exert synergistic antibacterial effects through multiple mechanisms. Membrane disruption: In Gram-positive bacteria, direct action on the cytoplasmic membrane; in Gram-negative bacteria, disruption of the outer membrane followed by action on the inner membrane. Both lead to membrane potential imbalance, content leakage, and altered permeability. Metabolic interference: Upon entry, PAs interfere with metabolic processes, inhibiting bacterial growth. This mechanism is similar in both bacterial types. Intracellular acidification: Upon entry, PAs dissociate and release H^+^, lowering intracellular pH. This mechanism is similar in both bacterial types.

The primary antimicrobial mechanism of PAs involves disrupting bacterial cell structure. This process is initiated by shifts in surface charge and cellular hydrophobicity, culminating in membrane destabilization, poration, and the subsequent efflux of essential intracellular constituents [34,59]. The microbicidal efficacy of PAs is intrinsically linked to their lipophilicity; typically, compounds with higher lipid solubility demonstrate a superior capacity to partition into and destabilize bacterial lipid bilayers. Structural modifications, such as the substitution of hydroxyl groups with methoxy moieties, can elevate molecular lipophilicity, thereby facilitating membrane insertion and augmenting antibacterial performance [60]. As shown in Figure 5, the antibacterial mechanisms of PAs differ based on bacterial cell wall morphology. In Gram-positive bacteria, which lack a protective outer membrane, PAs interact directly with the cytoplasmic membrane. Once inside the cytoplasm in their undissociated form, PAs dissociate, enhancing membrane permeability and compromising transport systems essential for ionic homeostasis, inducing a collapse of the membrane potential. Conversely, the efficacy of PAs against Gram-negative bacteria is largely contingent upon their undissociated form. In this state, specific PAs traverse the outer membrane via passive diffusion or through porin channels, with molecular dimensions serving as a key controlling factor. Once localized within the cytoplasm, PA dissociation triggers a multi-target cascade, lowering intracellular pH, denaturing functional proteins, and inducing massive K^+^ efflux, ultimately leading to metabolic arrest and cell death [61]. Furthermore, certain PAs like FA can intercalate into the phospholipid bilayer, physically obstructing the transmembrane transport of substrates important for cellular metabolism [61,62]. A systematic synthesis of 158 studies (2013–2025) has identified three converging antibacterial targets that operate in parallel, namely ROS generation (exhibited by 72% of tested phenolics), membrane disruption (58%), and DNA interaction (41%). Importantly, these targets are not mutually exclusive; instead, ROS can trigger lipid peroxidation that further compromises membrane integrity, facilitating phenolic uptake and accelerating DNA damage. This multi-target cascade, which has been documented in PAs among other phenolics, underlies the broad-spectrum efficacy of PAs and mitigates the risk of resistance development [63].

### 3.2. Antioxidant Mechanisms of PAs

The potent antioxidant capacity of PAs is primarily derived from the phenolic hydroxyl groups within their molecular structure, which serve as hydrogen atom donors to intercept or terminate oxidative radical chains [64]. Natural PAs exert their protective effects through three complementary pathways, namely direct free radical scavenging, the chelation of pro-oxidative transition metal ions, and the absorption of UV radiation (100 to 400 nm). While the first two constitute classical antioxidant mechanisms, UV absorption functions as a photoprotective effect, shielding sensitive food components from high-energy photons and thereby mitigating photo-oxidative deterioration. Collectively, these actions delay oxidative rancidity, suppress the evolution of off-flavors, and preserve food quality during storage [65]. Specifically, phenolic hydroxyl groups neutralize radicals via hydrogen atom donation, yielding resonance-stabilized phenoxyl radicals that inhibit the propagation of oxidative cycles. Molecules such as sinapic acid and FA exemplify this stabilization mechanism [41,66]. Additionally, CA and CGA, characterized by their catechol moieties, efficiently quench superoxide radicals through coordinated electron transfer and proton donation, effectively converting them into H_2_O_2_ [67].

### 3.3. Structure–Activity Relationships of PAs

**Figure 6:**
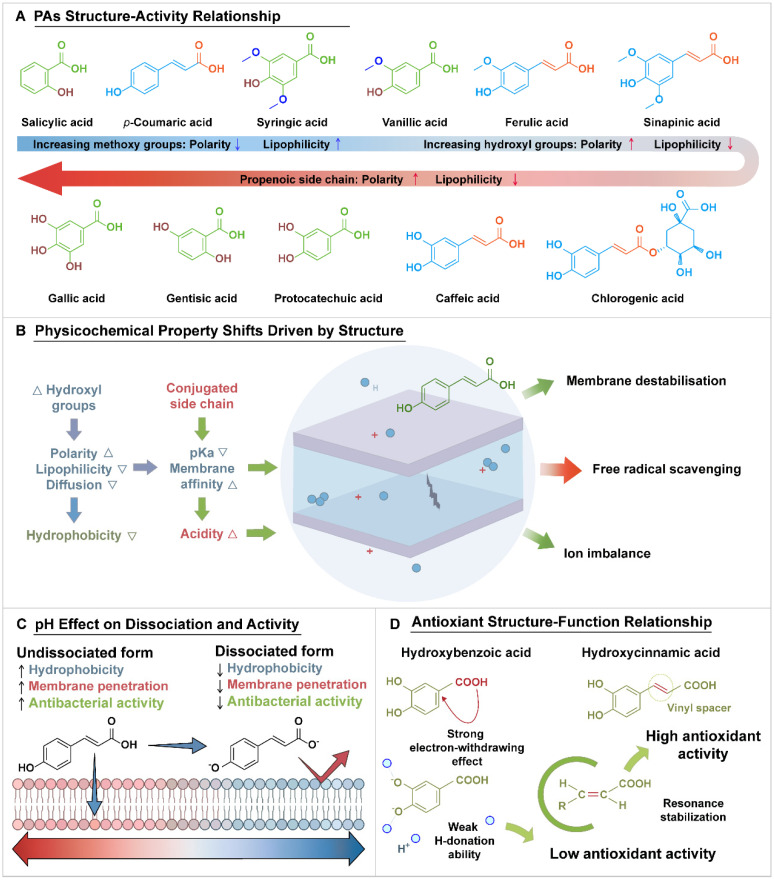
Structure–activity relationship of phenolic acids (PAs): Impact of substitution patterns on physicochemical properties and bioactivities [34,42].

The functional efficacy of PAs strictly depends on their molecular structure (Figure 6). Bioactivity is modulated by the density and positioning of hydroxyl and methoxy substituents, the electronics of acidic functional groups (e.g., carboxyls), and the degree of side-chain saturation [34,60]. Within the HBA series, antibacterial potency generally exhibits an inverse correlation with the number of hydroxyl groups [34]. As weak organic acids, both HBAs and HCAs act predominantly in their undissociated states to passively diffuse across bacterial envelopes. Subsequent intracellular dissociation leads to acidification, enzymatic inactivation, and ionic dysregulation. Consequently, physicochemical parameters such as the acid dissociation constant (pKa) and lipophilicity are decisive factors in bactericidal efficiency. HCAs often exhibit antimicrobial profiles superior to their HBA counterparts with equivalent hydroxyl counts. For example, the propenyl side chain of CA reduces molecular polarity compared to PCA, enhancing lipophilicity and membrane penetration [42]. In HCAs, antimicrobial performance is also strongly reinforced by conjugated double bonds in the side chain, which facilitate membrane interactions [34]. Regarding antioxidant structure–activity relationships, the electron-withdrawing nature of the carboxyl group in benzoic derivatives can diminish the hydrogen-donating capacity of adjacent hydroxyls. However, the insertion of a vinyl or methylene bridge, as seen in HCAs, mitigates this effect, typically resulting in enhanced radical-scavenging capacity relative to simple HBAs [34]. While hydroxyl density is fundamental, lipophilicity often emerges as the dominant determinant for antimicrobial efficacy, where increased hydrophobicity promotes deeper insertion into the phospholipid bilayer, triggering irreversible cellular leakage [68]. Furthermore, environmental pH modulates performance; acidic conditions suppress deprotonation, thereby increasing hydrophobicity and promoting deeper membrane intercalation [9,42].

### 3.4. Antioxidant Mechanisms of Phenolamides

Phenolamides exhibit pronounced antioxidant capacities, which underpin many of their documented bioactivities [56]. HCAATs demonstrate potent quenching of free radicals and ROS in various chemical assays. Research indicates that phenolamides conjugated with aromatic monoamines possess exceptional antioxidant potential; CA-derived phenolamides, for instance, are highly effective at inhibiting lipid peroxidation. Furthermore, dimeric phenolamides exhibit dual functionality: enhanced radical-scavenging and potent iron-chelating abilities, which interrupt metal-catalyzed oxidative cascades [45]. Antioxidant potency within CA-derived phenolamides typically follows the hierarchy: *N*-*trans*-caffeoyldopamine > *N*-*trans*-caffeoyltyramine > *N*-*trans*-caffeoyltryptamine. In dopamine-containing HCAAs, a previous study reported the activity trend as *N*-*trans*-caffeoyldopamine ≈ *N*-*trans*-cinnamoyldopamine > *N*-*p*-coumaroyldopamine > *N*-*trans*-feruloyldopamine > *N*-sinapoyldopamine [44]. Substituents significantly influence these trends; hydroxyl groups on the phenolic ring facilitate direct oxidation and stabilize resulting radicals, while methoxy substitutions generally attenuate activity [44]. Comparative data suggest the caffeoyl moiety is the primary antioxidant driver, while dopamine serves as the optimal amine conjugate. The synergy between PAs and polyamines, both inherently antioxidant, results in the amplified efficacy of their amide conjugates [44,56].

Mechanistically, based on structural analogy with PAs, phenolamides may employ electron-rich functional groups to coordinate with Fe^2+^, Fe^3+^, and Cu^2+^, forming stable metal-ligand complexes that prevent the generation of reactive radicals [69]. DFT analyses reveal that solvent polarity is a critical modulator of the antioxidant mechanism. In nonpolar environments, phenolamides scavenge radicals via HAT, whereas in polar media, the mechanism shifts to pH-dependent mixed pathways, such as SPLET [45].

### 3.5. Antimicrobial Mechanisms of Phenolamides

In stark contrast to PAs, the antimicrobial mechanisms of phenolamides under conditions relevant to food preservation remain a profound knowledge gap. The current evidence base is almost exclusively derived from contexts outside of food science, and direct experimental validation in food matrices or against common foodborne pathogens is entirely absent.

Molecular docking simulations suggest potential intracellular targets. For instance, *N*-caffeoyltyramine was predicted to inhibit *Helicobacter pylori* isoleucyl-tRNA synthetase (IleRS) with favorable binding affinity, which could theoretically block protein synthesis [70]. However, the relevance of this clinically oriented finding to, for example, the inhibition of *Listeria monocytogenes* on a ready-to-eat meat surface is unknown.

To date, the antimicrobial activities of phenolamides have been characterized primarily through in vitro susceptibility assays (e.g., MIC determinations against various bacteria and fungi) and phenotypic observations in plant and model food systems [58,71]. However, only one study has experimentally elucidated a specific molecular target and complete mechanism of action for a phenolamide. Pisithkul et al. [72] demonstrated that feruloyl amides and coumaroyl amides act as potent, fast-acting competitive inhibitors of glutamine PRPP (5-phosphoribosyl-1-pyrophosphate) amidotransferase in *Escherichia coli*, thereby blocking de novo purine and pyrimidine biosynthesis. Within 10 min of exposure, these compounds induced a rapid accumulation of PRPP and a sustained reduction in carbon and nitrogen flux into nucleotides, an effect fully rescued by exogenous nucleoside supplementation.

However, the translational relevance of this finding to food preservation is limited and must be interpreted with caution. The study was conducted in the context of lignocellulosic hydrolysate toxicity during biofuel fermentation, a chemically harsh, nutrient-limited environment that bears little resemblance to the high-nutrient, near-neutral pH conditions typical of most perishable foods. Moreover, the test organism was a laboratory strain of *Escherichia coli* adapted to industrial stress, not a foodborne pathogen such as *Listeria monocytogenes* or *Staphylococcus aureus* growing on meat or produce surface. Whether phenolamides exert the same nucleotide biosynthesis inhibition in foodborne pathogens, and whether this mechanism remains operative in the presence of exogenous nucleosides abundant in food matrices (e.g., from hydrolyzed proteins or yeast extracts), is entirely unknown. Therefore, while nucleotide biosynthesis inhibition represents a plausible hypothesis for further investigation, it cannot yet be considered an established antimicrobial mechanism of phenolamides under food-relevant conditions.

Despite the growing catalog of phenolamides with documented antimicrobial activity, mechanistic investigations remain strikingly scarce. A recent chemoenzymatic study by Dozio et al. [71] demonstrated differential antibacterial effects of feruloyl amides against Gram-positive and Gram-negative bacteria. However, the work was centered on anti-appressorium activity in plant pathogens and did not probe the molecular basis for the observed antimicrobial phenotypes. Collectively, the available evidence underscores that no unified or food-relevant mechanism of action has been established for this compound class.

Therefore, rather than presenting a defined mechanism of action, this section highlights a critical frontier for future research. Unraveling the antimicrobial mode of action of phenolamides in food-relevant models is a prerequisite for their rational application as targeted preservatives. Table 3 provides a consolidated evidence classification for the antimicrobial and antioxidant mechanisms discussed in Section 3.1, Section 3.2, Section 3.3, Section 3.4 and Section 3.5.

## 4. Application Strategies of PAs and Phenolamides in Food Preservation

This section focuses on the technological principles and design rationales underlying various delivery strategies for PAs and phenolamides. The emphasis is on how each strategy functions at the physicochemical level, controlled release kinetics, interfacial partitioning, and barrier properties, rather than on their performance in specific food products. Section 5 subsequently provides a comparative evaluation of how effectively these strategies perform across different food matrices, with an emphasis on shelf-life extension and quality metrics. This two-part structure allows a clear separation between the engineering of delivery systems and their validation in complex food environments.

Phenolic compounds are important natural functional additives with extensive applications in food preservation. Their primary functionalities include color stabilization, microbial growth inhibition, and the suppression of lipid oxidation, which collectively extend the shelf life of food products [37]. However, despite their potent bioactivity, the practical application of natural phenolics is hindered by several constraints. They exhibit inherent instability when exposed to common food processing and storage stressors, such as elevated temperatures, light, and oxygen. Furthermore, their susceptibility to degradation and relatively low aqueous solubility significantly diminish their bioavailability within complex food systems [64]. The efficacy of phenolics is inextricably linked to the properties of the food matrix. Physicochemical parameters, such as pH, lipid content, and water activity, alongside biological factors like the indigenous microflora, govern their antimicrobial and antioxidant performance. Notably, the bioactivity observed in situ is frequently substantially lower than in vitro measurements, as interactions with proteins, partitioning into lipid phases, and buffering effects can mask up to 90% of the intrinsic activity [63]. Recent studies have begun to characterize specific interactions. For instance, whey protein–pectin complexes have been shown to sequester PAs such as RA and CGA via hydrogen bonding, with binding affinities dictated by pH and phenolic structure [73]. Whether these interactions follow predictable thermodynamic or kinetic laws remains a critical knowledge gap. Encouragingly, emerging deep learning models have demonstrated potential in predicting polyphenol–protein binding affinities using structural and kinetic descriptors [74]. Adapting such predictive modeling for PAs represents a promising frontier for rational formulation design. An overview of these representative delivery and application strategies for PAs in food systems is presented in Table 4.

### 4.1. Direct Incorporation

Direct incorporation is the most conventional strategy, involving the integration of antimicrobial PAs or related bioactive compounds directly into food formulations [65]. By physically homogenizing PAs with other ingredients, the growth of pathogenic and spoilage microorganisms can be inhibited. In liquid or semi-solid systems, this is typically achieved through mechanical agitation, whereas solid food surfaces are treated by dipping or spraying with PA solutions [79]. Although operationally straightforward, this approach possesses distinct limitations. Achieving uniform distribution in heterogeneous matrices is difficult, and maintaining antimicrobial concentrations at contamination-prone sites (e.g., the food-air interface) is often infeasible, thereby reducing overall preservation efficacy [80].

### 4.2. Encapsulation Strategies

Encapsulation has emerged as an effective strategy to circumvent low bioavailability, chemical instability, and non-target interactions of PAs. This technique involves entrapping active compounds within carrier materials to form micro- or nanoscale barriers. Encapsulation optimizes spatial distribution, shields PAs from oxidative stressors, and enhances functional longevity. A primary advantage of this strategy is the capacity for controlled release. Release kinetics can be engineered to respond to environmental stimuli, such as pH fluctuations, enzymatic activity, or temperature shifts, enabling site-specific delivery [81]. For example, embedding antioxidants within packaging structures allows for gradual migration toward the food surface, maintaining a stable antioxidative microenvironment [65]. Commonly employed physicochemical techniques for encapsulation include spray drying, electrospinning, liposome preparation, and solvent precipitation [81]. The selection of wall materials is critical for determining the performance and regulatory compliance of the final product. For hydrophilic PAs, natural polymers such as polysaccharides (e.g., starch, alginate, xanthan gum) and proteins (e.g., gelatin, caseins) are preferred [82].

### 4.3. Edible Films and Coatings

Edible films and coatings involve the incorporation of PAs into biopolymer matrices applied directly to food surfaces, creating a multi-functional barrier. While edible films are pre-formed standalone sheets, coatings are generated in situ through dipping or spraying followed by solvent evaporation. These systems effectively restrict the ingress of oxygen and moisture while inhibiting microbial colonization. Common matrix materials include proteins, polysaccharides, lipids, or their composites [83]. Chitosan (CS) has garnered significant interest as a matrix material due to its intrinsic antimicrobial properties and excellent film-forming capacity. However, simple physical blending often results in rapid, burst-release profiles. To address this, phenolic acid-grafted chitosan (PA-g-CS) has been developed. This strategy covalently anchors phenolic moieties to the chitosan backbone, enhancing both stability and long-term bioactivity [2,64,83].

Current grafting techniques include carbodiimide-mediated coupling, free-radical-mediated grafting, enzymatic catalysis, and ion exchange, each influencing the structural characteristics and subsequent bioactivity of the conjugate [2,84]. Notably, the bioactivity of PA-g-CS is primarily driven by the intrinsic structure of the grafted PA; for instance, CA-g-CS often exhibits superior DPPH radical scavenging activity compared to FA-g-CS due to the ortho-dihydroxy (catechol) moiety, which facilitates hydrogen atom donation [84,85].

Despite these promising preservation benefits, a critical safety question regarding the fate of these grafted materials upon ingestion remains completely unexplored. Given direct food contact, consumers inevitably ingest small amounts of PA-g-CS. While in vitro cytotoxicity tests have reported no acute toxicity for PA-g-CS [2], such assays cannot predict chronic low-level ingestion effects, bioaccumulation, gut-microbiome interactions, or potential release of PAs in the lower intestine. Therefore, future studies should employ simulated gastrointestinal digestion models coupled with liquid chromatography-tandem mass spectrometry to track the fate of amide linkages, identify digestion products, and conduct comparative toxicity assays between the intact conjugates and their potential breakdown products.

### 4.4. Active Packaging

Active packaging is an advanced strategy designed to dynamically maintain food quality through the controlled release of antioxidants or the absorption of harmful substances. Unlike conventional “passive” packaging, active systems integrate functional components like oxygen scavengers or antimicrobial agents [40]. PAs can be utilized in active packaging through three primary modes: direct embedding into the polymer matrix for controlled migration, incorporation into sachets for headspace diffusion, or chemical immobilization on the inner surface for contact-active antimicrobial effects [78].

Advanced material science, such as the development of polymer nanocomposites, allows for the controlled and, in some advanced systems, stimuli-responsive release of active compounds. For example, intercalating RA and salicylic acid into Mg-Al layered double hydroxides within polyethylene films has demonstrated potent antimicrobial activity [1]. These innovations highlight the transition toward sustainable, high-performance “green” packaging solutions.

## 5. Applications of PAs in Different Food Systems

Whereas Section 4 addressed the design principles of PA delivery systems, this section evaluates their comparative performance in real food matrices. The discussion is organized by food commodity type, covering fresh produce, meat, aquatic products, and lipid-rich emulsified systems, followed by a dedicated subsection on packaging systems as integrated application platforms (Section 5.5). Table 5 provides a consolidated overview linking dominant deterioration pathways to preservative mechanisms across these matrices.

### 5.1. Preservation of Fruits and Vegetables

Postharvest fruits and vegetables are metabolically active tissues. Due to their delicate structural integrity and high water activity, they undergo persistent respiration and complex physiological transitions during storage, leading to rapid quality senescence. This deterioration is manifested through moisture transpiration, nutrient depletion (e.g., vitamins, minerals, and carbohydrates), and enzyme-mediated softening, browning, and flavor degradation [2,90]. Furthermore, spoilage involves a synergistic interplay between physiological senescence and microbial infection. Microbiological threats from spoilage microflora and foodborne pathogens, such as *Listeria monocytogenes*, represent major challenges to the shelf-life extension of fresh produce [2]. Consequently, the inherent perishability of these commodities results in substantial postharvest economic losses, necessitating effective preservation strategies that simultaneously delay senescence and microbial proliferation.

In both solution immersion and surface coating applications, PA-g-CS systems offer significant functional advantages. For instance, grapefruits treated with 10 mg/mL salicylic acid-g-CS via immersion exhibited potent suppression of green mold (*Penicillium digitatum*) while maintaining textural firmness. This protective effect is attributed to the coating’s capacity to inhibit the degradation of cell wall polysaccharides, thereby retarding tissue softening [2]. Similarly, CGA-g-CS coatings have outperformed native CS in reducing weight loss and respiration rates in apricots and peaches, while preventing undesirable fluctuations in soluble solids and titratable acidity [3].

Immersion treatment of cherry tomatoes with GA-g-CS inhibited enzymatic browning and preserved the integrity of the ascorbate-glutathione cycle. Compared to physical blends of salicylic acid and CS, the covalently grafted salicylic acid-g-CS coatings more effectively up-regulated endogenous antioxidant enzymes, such as superoxide dismutase and catalase, thereby enhancing the fruit’s internal stress resistance [2]. For highly perishable berries, CA-g-CS coatings proved superior in preserving anthocyanins, total phenolics, and ascorbic acid [2,90]. In mushroom preservation, GA-g-CS films maintained higher phenolic content and enzymatic activities compared to commercial polyethylene films [3].

Beyond direct coatings, active packaging platforms, such as GA/chitin nanofiber composite films, GA-phycocyanin/PVA active films, and FA/gelatin photoactive coatings, have demonstrated remarkable efficacy in suppressing fungal pathogens and extending the shelf life of berries and grapes [86,91,92]. A detailed cross-category comparison of these packaging systems, including their release kinetics and preservation mechanisms, is presented in Section 5.5. Collectively, the primary role of PAs in produce systems is the dual-action inhibition of fungal infection and the mitigation of metabolic imbalances.

### 5.2. Preservation of Meat Products

Meat products provide an ideal substrate for microbial growth and oxidative cascades. Outbreaks of foodborne illness associated with contaminated refrigerated meat are a global concern, as meat remains vulnerable to pathogens even under chilled storage. Thus, the development of natural preservatives with synchronized antimicrobial and antioxidant activities is a critical objective [3]. Beyond microbial issues, lipid oxidation is a primary factor limiting commercial value. Meat is rich in unsaturated fatty acids and proteins that undergo autoxidation, forming primary hydroperoxides and secondary volatile metabolites (e.g., aldehydes and ketones) [5,90]. These reactions induce rancidity, discoloration, and the formation of potentially carcinogenic oxidation products. To combat this, the inclusion of antioxidants is essential. While synthetic additives like butylated hydroxytoluene and butylated hydroxyanisole are common, evidence links their metabolites to endocrine disruption and DNA damage [8].

Treatment of fresh pork with CA-g-CS or *p*-coumaric acid-g-CS (10 mg/mL) effectively delayed microbial proliferation and the formation of thiobarbituric acid reactive substances (TBARS) [2]. GA-g-CS coatings extended the refrigerated shelf life of pork from 6 to 18 days by stabilizing appearance and oxidative markers [3,90]. FA-g-CS coatings reduced total volatile basic nitrogen (TVB-N) and drip loss, extending shelf life by approximately 7 days [90]. In addition to direct coating strategies, active packaging films, such as FA-loaded nanoemulsion-reinforced CS films and starch-polyester bilayer films, have been successfully applied to extend the shelf life of packaged pork through controlled release of PAs [40,77] (see Section 5.5 for a systematic comparison of these and other packaging platforms). In meat systems, the efficacy of PAs is fundamentally driven by their capacity to terminate free radical chain reactions.

### 5.3. Preservation of Aquatic Products

Aquatic products are exceptionally perishable due to their high moisture content and endogenous enzyme activity. Postmortem spoilage involves rapid protein degradation, lipid oxidation, and the evolution of volatile bases such as trimethylamine [90].

Studies demonstrate that GA-g-CS (1%), combined with vacuum packaging, maintains microbial community balance and selectively inhibits spoilage bacteria in sea bass (*Lateolabrax japonicus*) fillets [93]. In white shrimp (*Penaeus vannamei*), CGA-g-CS coatings significantly attenuated weight loss and TVB-N accumulation [2]. PA-g-CS coatings have extended the shelf life of Japanese sea bass by more than 6 days, golden pompano (*Trachinotus ovatus*) fillets by at least 9 days, and shrimp by approximately 8 days [3]. Active packaging pads based on CS-GA/PVA hydrogels have also been developed to mitigate myofibrillar protein oxidation in fish fillets [88], with their design principles and comparative performance discussed in Section 5.5. Notably, the role of PAs in aquatic systems is more pronounced in the regulation of protein oxidation compared to terrestrial meat.

### 5.4. Preservation of Lipid-Rich and Emulsified Food Systems

Oil-rich foods are highly susceptible to oxidative rancidity through the autoxidation of unsaturated fatty acids, generating toxic secondary metabolites [94]. In emulsified systems, this process is complicated by the oil–water interface, which determines antioxidant partitioning [89].

In low-moisture lipid systems, the antioxidant action of PAs relies primarily on the direct interception of peroxyl radicals within the bulk lipid phase. Simsek et al. evaluated CGA from green coffee extract in hazelnut paste stored at 4, 25, and 40 °C for three months. Based on the regression equation of the oleic acid/linoleic acid ratio, the predicted shelf life of the control at 22 °C was 28 days, which extended to 60 days with 0.5% green coffee extract and 90 days with 0.75% green coffee extract [95]. Similarly, GA-g-CS coatings effectively suppressed peroxide value and TBARS formation in peanut powder and corn oil, exhibiting a DPPH scavenging rate of 89.5% compared to only 9.4% for unmodified CS [3]. These examples demonstrate that in low-moisture matrices, direct radical scavenging and surface barrier effects are the predominant protective mechanisms.

In emulsions, however, interfacial activity is critical. Qiu et al. [89] covalently modified egg yolk phospholipids with three cinnamic acid-derived PA, namely FA, CA, and *p*-coumaric acid, to produce multifunctional phospholipid derivatives that serve as both emulsifiers and antioxidants. These derivatives reduced droplet size from 394.60 to 238.09 nm while simultaneously inhibiting lipid oxidation. Among them, CA-modified phospholipid (CA-PL) achieved the greatest reduction in malondialdehyde formation (62.3% after 28 days). This can be attributed to the potent antioxidant activity of the CA moiety. As discussed in Section 3.4, the catechol group of CA facilitates efficient hydrogen donation and radical stabilization, whereas the methoxy group of FA enhances lipophilicity but generally attenuates antioxidant activity [42]. Notably, FA-PL exhibited greater stability under alkaline conditions, and *p*-coumaric acid-PL offered higher synthesis efficiency, illustrating that PA selection should be tailored to the specific emulsion environment.

Collectively, these studies underscore a fundamental distinction. In low-moisture pastes and powders, direct contact between PAs and the lipid phase governs efficacy, whereas in emulsions, the interfacial partitioning determined by the hydrophilic-lipophilic balance governs antioxidant performance. No single PA is universally optimal across all matrices; rather, delivery strategies and compound selection must be guided by the dominant oxidation pathway and the physicochemical nature of the food system.

### 5.5. Packaging Systems as Integrated Application Platforms

Unlike the food-category-specific discussions above, this section provides a cross-cutting analysis of packaging systems as integrated platforms for PA delivery. Packaging systems warrant a dedicated subsection because, unlike direct addition, encapsulation, or edible coatings, their application spans multiple food categories and their performance is highly matrix-dependent, making cross-category comparison essential for guiding rational design. By comparing their design principles and performance across produce, meat, and aquatic products, we aim to identify the common and distinct requirements that different food matrices impose on active packaging.

In fresh produce, Cabrera-Barjas et al. [91] developed GA/chitin nanofiber films that achieved >95% inhibition of Botrytis cinerea in strawberries, while Gong et al. [86] used a GA-phycocyanin/PVA film to extend grape shelf life to 13 days and blueberry shelf life to 20 days. Furthermore, Shi et al. [92] developed a FA/gelatin photoactive coating that achieved >98.5% inactivation of pathogens under light exposure. These systems primarily function by creating a modified atmosphere and providing sustained antifungal activity, addressing the dual challenge of microbial infection and metabolic senescence in postharvest tissues. In meat products, FA-loaded nanoemulsion-reinforced CS films suppressed protein degradation and lipid oxidation, extending pork shelf life by 6 days [77], and starch-polyester bilayer films effectively reduced lactic acid bacteria counts in packaged pork [40]. Here, the packaging must serve as a strong oxygen barrier and provide lipophilic antioxidant delivery, as lipid oxidation and protein degradation are the dominant deterioration pathways in muscle foods. In aquatic products, CS-GA/PVA hydrogel preservation pads mitigated myofibrillar protein oxidation in sea bass [88], highlighting the need for moisture control and specific protein protection in high-water-activity environments, where both microbial spoilage and endogenous enzyme activity accelerate quality loss.

A comparative analysis of these packaging systems across food categories reveals distinct design–function relationships. In produce, the priority is sustained antifungal activity within a modified atmosphere, as the high water activity and respiration of fresh tissues demand both microbial control and gas exchange. In muscle foods, by contrast, the low oxygen permeability of the packaging and the lipophilic nature of the antioxidant delivery are paramount, because lipid oxidation propagates via radical chain reactions within the hydrophobic phase. Aquatic products introduce a further complexity: the combination of high moisture, active endogenous proteases, and rapid microbial growth necessitates packaging that simultaneously absorbs excess liquid and protects myofibrillar proteins from oxidative denaturation. These divergent requirements explain why no single packaging design is universally optimal. Hydrogel-based absorbent pads are well suited to high-moisture aquatic products, whereas oxygen-barrier films with lipophilic antioxidant delivery are more appropriate for muscle foods.

## 6. Barriers to the Application of Phenolamides in Food Preservation

As documented in the preceding section, the application of PAs in diverse food systems is supported by extensive literature. The conspicuous absence of parallel examples for phenolamides in Section 5 is not an oversight; it accurately reflects the nascent state of this field. Despite the potent antimicrobial and antioxidant activities of phenolamides reported in numerous in vitro studies using laboratory-scale purified compounds, their translation into practical food preservation is still at an early stage. To date, most research has utilized crude plant extracts rather than isolated molecules, leaving the direct application of pure phenolamides as primary preservatives in complex perishable matrices. A notable exception is the recent work by Van Zadelhoff et al. [58], who demonstrated that phenolamide dimers from barley rootlets exhibit significant anti-yeast activity in alcohol-free beer. This study provides an important proof-of-concept for their functionality in a liquid food system. However, a substantial gap persists between these controlled findings and the systematic deployment of phenolamides in solid or semi-solid foods, such as fresh meat or produce.

A fundamental bottleneck is the low natural abundance of phenolamides relative to simpler PAs. Although specific sources like oats and barley rootlets can accumulate these compounds, isolating milligram-scale quantities of high-purity monomers requires exhaustive rounds of preparative chromatography, which is economically prohibitive [10]. This scarcity forces a reliance on crude or enriched extracts (e.g., from tomato leaves), where bioactivity cannot be definitively ascribed to phenolamides [96]. In such mixtures, the presence of co-existing flavonoids, hydroxycinnamates, and other phenolics often masks the true active agents, creating a confounding variable in mechanistic studies. The lack of pure standards prevents rigorous dose–response and mechanistic modeling, which in turn stifles industrial investment in large-scale purification or biotechnological synthesis.

Furthermore, the direct application of specific phenolamides, such as avenanthramide C, is hindered by intrinsic physicochemical and biopharmaceutical limitations. As highlighted by Sen et al. [97], these compounds often exhibit poor aqueous solubility and limited stability within the gastrointestinal tract, which correlates with poor dispersibility in aqueous food systems and degradation during thermal processing or extended shelf life. Moreover, free (unencapsulated) avenanthramide C exhibits a bioaccessibility of less than 10% during simulated digestion, suggesting its biological efficacy may be compromised in complex food environments. While nano-delivery systems, such as those utilizing barley-derived self-assembling peptides, can enhance bioaccessibility to nearly 50%, these strategies face significant hurdles, including sophisticated manufacturing requirements, stringent regulatory scrutiny, and release profiles that may not align with the immediate antimicrobial action required on food surfaces.

Finally, the historical focus of phenolamide research has been dominated by pharmacology (e.g., anti-inflammatory, and neuroprotective effects) and plant pathology (as phytoalexins), rather than food science [56,57]. Consequently, the food science community has developed a “path dependence” toward simpler PAs, resulting in a well-established infrastructure of analytical protocols and commercial products. While this infrastructure has been instrumental in advancing PA applications, it inadvertently creates a barrier for phenolamides, whose greater structural diversity and amphiphilic nature do not easily fit within existing frameworks [11,44].

## 7. Safety Evaluation of PAs and Phenolamides

Safety remains the primary criterion for incorporating any compound into food systems. Compared to volatile antimicrobial agents like essential oils, PAs offer a milder sensory profile and lower volatility while maintaining comparable efficacy, making them ideal food additives [7,34]. Structure–activity relationship analyses have examined the cytotoxic potential of major dietary PAs, revealing that this activity is influenced by the number and position of hydroxyl groups [42]. For example, RA exhibits negligible toxicity toward human lymphocytes, as it does not trigger excessive ROS or apoptotic pathways [98]. Similarly, CA has shown no cytotoxicity toward macrophage cell lines at concentrations up to 100 μg/mL [99]. Many PAs also exhibit low cytotoxicity toward normal mammalian cells at concentrations relevant to food applications, which underscores their potential as safe food preservatives [42,98].

The safety profile of phenolamides is also becoming clearer, albeit primarily from pharmacological or nutraceutical perspectives [56]. Lee et al. [100] reported a no-observed-adverse-effect level of 1427–1983 mg/kg/day for *N*-*trans*-caffeoyltyramine in a 90-day rodent study, supporting its safety as a dietary ingredient. This safety assessment should be extended to address food-matrix interactions and thermal stability, which are standard requirements for direct food additives. Such studies represent a logical next step. Additionally, Roumani et al. [96] confirmed that major tomato leaf phenolamides lack significant cytotoxicity toward THP-1-derived macrophages. Furthermore, the long dietary history of oat-derived avenanthramides offers a reassuring baseline of human exposure, and ongoing efforts to align these naturally occurring compounds with established food additive regulatory frameworks will further enable their commercial application in food preservation [50].

Despite these encouraging safety profiles, several critical gaps must be addressed before PAs and phenolamides can gain widespread regulatory approval as direct food additives. First, estimated dietary intake assessments remain largely unavailable. Although PAs are naturally present in many plant-based foods, the intake levels resulting from their deliberate addition as preservatives have not been systematically modeled. Exposure assessments based on proposed use levels across different food categories are a prerequisite for regulatory submission in most jurisdictions. Second, thermal stability during food processing has received limited attention. Many phenolic compounds are susceptible to thermal degradation during pasteurization, sterilization, or baking. However, systematic studies on the identity, yield, and safety of their degradation products under realistic processing conditions remain sparse. For phenolamides, no data exists on their thermal degradation pathways. Third, while selected phenolic-rich preparations such as rosemary extract (E 392), which is rich in PAs including rosmarinic acid, have obtained regulatory approval in the EU [101], the regulatory pathway for phenolamides remains undefined. The absence of standardized analytical methods, compositional specifications, and chronic toxicological data constitutes a significant barrier to regulatory acceptance.

## 8. Conclusions and Perspectives

Significant strides have been made in establishing plant-derived PAs as effective and safe natural preservatives. This review has described their classification, structural attributes, and multi-target antimicrobial and antioxidant mechanisms. Key structure–activity relationships indicate that the ortho-dihydroxy (catechol) motif is essential for superior antioxidant capacity, while methoxylation modulates lipophilicity to enhance membrane permeability. To mitigate the instability of free PAs in food matrices, advanced delivery platforms, including nano-encapsulation, chitosan-grafted edible films, and active packaging, have proven successful in maintaining efficacy across seafood, meat, and produce.

However, the systematic application of phenolamides in food preservation remains underdeveloped. To transition these compounds from laboratory research to industrial application, future research must prioritize mechanistic studies by utilizing computational simulations (e.g., DFT, molecular docking, and molecular dynamics simulation) alongside experimental determination to elucidate structure–activity relationships. Additionally, future work should focus on scalable production by leveraging engineered microbial cell factories to generate high-purity, food-grade phenolamides in a cost-effective manner. In situ validation is also essential, which means conducting preservation trials in real-world perishable food matrices to measure shelf-life extension under realistic storage conditions. Furthermore, regulatory compliance must be achieved by completing the full toxicological assessment, from in vitro assays to chronic in vivo studies, so that safe dosage limits can be reliably established.

A broader methodological consideration merits attention. While in situ efficacy application studies of PAs across real food matrices are now abundant, including in meat, seafood, produce, and emulsion systems, detailed mechanistic investigations at the molecular level have largely been confined to laboratory model strains and idealized conditions. Bridging this gap between empirical efficacy and mechanistic understanding remains a shared challenge that future work across both compound classes must address.

Beyond individual use, exploring synergistic systems that combine PAs with phenolamides, or integrating them with emerging physical technologies like cold plasma and UV treatment, represents a promising frontier. Addressing these challenges could position phenolamides as promising candidates within the next generation of natural food preservatives. Their ultimate role, however, will be contingent upon the successful resolution of the scientific and industrial bottlenecks identified in this review.

## Figures and Tables

**Table 1:** Chemical information, dietary sources, and food-relevant bioactivities of major PAs.

	No.	Compound Name	Chemical Structure	CAS No.	Dietary Sources	Biological Activities	References
Hydroxybenzoic acids	1	Salicylic acid	_ 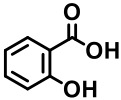 _	69-72-7	Fruits, Vegetables, Herbs, Spices	Antibacterial, Antifungal	[12]
	2	Protocatechuic acid	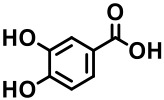	99-50-3	Fruits, Vegetables	Antibacterial	[13]
	3	Gallic acid	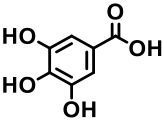	149-91-7	White tea, Black tea, Mango, Banana, Berries, Clove, Thyme, Chestnut	Antibacterial, Antioxidant, Antifungal	[14]
	4	Ellagic acid	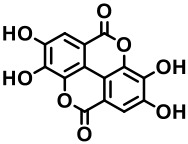	476-66-4	Pomegranates, Strawberries, Blackberries, Raspberries, Blueberries, Nuts, Seeds, Green tea	Antibacterial, Antioxidant	[15]
Hydroxycinnamic acids	1	*p*-Coumaric acid	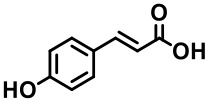	501-98-4	Tomatoes, Carrots, Cereals	Antibacterial, Antioxidant, Antifungal	[16]
	2	Caffeic acid	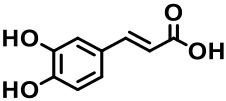	331-39-5	Coffee, Mint, Oregano, Rosemary, Thyme, Coriander, Cardamom, Blueberry, Yerba mate, Mango, Banana	Antibacterial, Antioxidant, Antifungal	[3]
	3	Ferulic acid	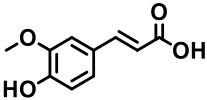	1135-24-6	Grasses, Grains, Vegetables, Flowers, Fruits, Leaves, Beans, Coffee bean, Artichoke, Peanut, Nuts	Antibacterial, Antioxidant, Antifungal	[17]
	4	Sinapinic acid	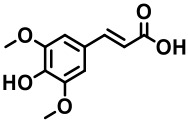	530-59-6	Rye, Fruits, Vegetables	Antibacterial, Antioxidant, Antifungal	[18]
	5	Chlorogenic acid	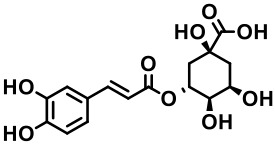	327-97-9	Apple, Peach, Pineapple, Blueberry, Coffee, Sunflower	Antibacterial, Antioxidant	[19]
	6	Rosmarinic acid	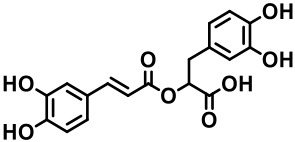	20283-92-5	*Malva sylvestris*, *Melissa officinalis*, *Salvia officinalis*, *Rosmarinus officinalis*, *Coleus blumei*	Antibacterial, Antioxidant, Antifungal	[20]

**Table 2:** Chemical information, dietary sources, and food-relevant bioactivities of major phenolamides.

	No.	Compound Name	Chemical Structure	CAS No.	Dietary Sources	Biological Activities	References
Phenolamides	1	Avenanthramide A	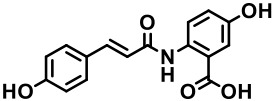	108605-70-5	Oat	Antioxidant	[21]
	2	Avenanthramide C	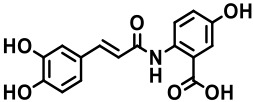	116764-15-9	Oat	Antioxidant	[22]
	3	*N*-*p*-*trans*-Coumaroyltyramine	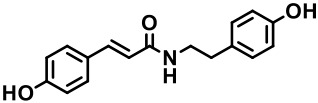	36417-86-4	*Solani melongenae* radix, *Corydalis edulis*, *Peperomia tetraphylla*, *Annona cherimola*, *Tribulus terrestris*, *Annona montana*, Chinese yam, Garlic, Welsh onion	Antibacterial, Antioxidant	[23]
	4	*N*-Caffeoyltyramine	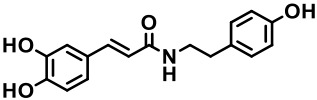	103188-48-3	*Annona crassiflora* seeds, *Annona montana*, *Annona cherimola*, *Lycium chinense*, *Vitis trifolia*, Hemp	Antibacterial, Antioxidant	[24]
	5	*N*-*p*-*trans*-Coumaroyloctopamine	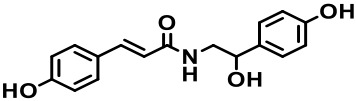	66648-45-1	*Lycianthes biflora*, *Phellodendron chinense*, *Celtis occidentalis*, *Lycium chinense*, *Polygonatum odoratum*, Eggplant, Garlic skin	Antioxidant	[25]
	6	*N*-*trans*-Caffeoyloctopamine	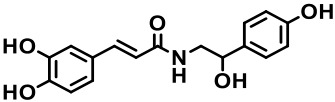	1378868-10-0	*Solanum melongena* L.	Antioxidant	[26]
	7	*N*-*trans*-Caffeoyldopamine	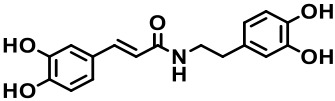	103188-49-4	*Capsicum annuum*, *Theobroma cacao*, *Lycium chinense*	Antibacterial, Antioxidant, Antifungal	[27]
	8	*N*-*trans*-Feruloyltyramine	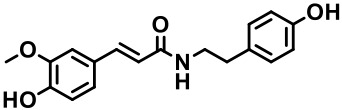	65646-26-6	*Balanites aegyptiaca*, *Hibiscus tiliaceus*, *Litsea greenmaniana*, *Polygonum hyrcanicum*, *Corydalis edulis*, *Cornulaca monacantha*, Potato, Sugar beet	Antioxidant, Antibacterial, Antifungal	[28]
	9	*N*-*trans*-Feruloyloctopamine	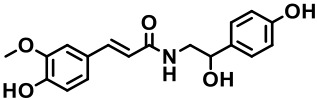	66648-44-0	*Melochia umbellata*, *Portulaca oleracea* L., *Polygonatum odoratum*, *Celtis occidentalis* L., Garlic skin, Potato	Antioxidant	[29]
	10	*N*-*trans*-Feruloyldopamine	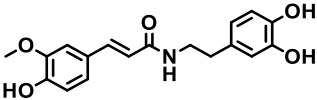	142350-99-0	*Corydalis impatiens*, *Arundo donax* L., *Atraphaxis spinosa* L.	Antioxidant	[30]
	11	*N*-Coumaroylserotonin	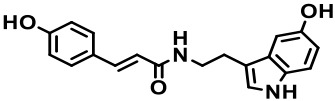	68573-24-0	*Centaurea*, Japanese barnyard millet, Safflower, Konnyaku	Antioxidant	[31]
	12	*N*-*trans*-Sinapoyltyramine	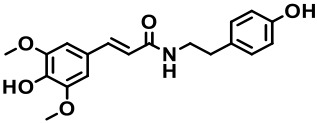	200125-11-7	*Peperomia tetraphylla*, *Porcelia macrocarpa*, *Tetragonia tetragonioides*, *Corydalis edulis*, *Corydalis impatiens*, *Lindera glauca*, *Amaranthus* spp., *Haloxylon articulatum*, Shallot	Antibacterial	[30]
	13	Di-feruloylputrescine	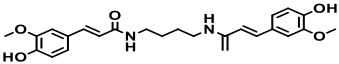	42369-86-8	Corn bran, Mousouchiku	antioxidant	[32]
	14	Lyciumamide A	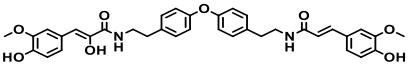	1647111-40-7	*Lycium barbarum*	Antioxidant	[33]

**Table 3:** Evidence classification for antimicrobial and antioxidant mechanisms of PAs and phenolamides.

Category	Mechanism	Compound Class	Evidence Tier	Food-Matrix Evidence	References
Antimicrobial	Membrane disruption	PAs (ellagic acid)	Experimentally confirmed	No	[59,61]
	Intracellular acidification	PAs	Probable	No	[61]
	ROS generation	PAs (gallic acid)	Experimentally confirmed	No	[63]
	DNA interaction	PAs (*p*-Coumaric acid, gallic acid)	Experimentally confirmed	No	[63]
	Membrane disruption	Phenolamides	Hypothetical	No	[71]
	Nucleotide biosynthesis inhibition	Phenolamides	Experimentally confirmed (*Escherichia coli* K-12, not food-isolated)	No	[72]
	IleRS inhibition	Phenolamides	Hypothetical (molecular docking)	No	[70]
Antioxidant	Hydrogen Atom Transfer	PAs	Hypothetical (DFT)	No	[66,67]
	Metal chelation	PAs (gallic acid)	Experimentally confirmed	No	[65]
	Hydrogen Atom Transfer	Phenolamides	Hypothetical (DFT)	No	[45]

Notes: Experimentally confirmed = direct measurement of the mechanism process itself. Probable = indirect experimental evidence from direct downstream consequences of the mechanism. Hypothetical = computational, structural, or correlative inference without mechanism-specific experimental validation. Food-matrix evidence indicates that the mechanistic evidence (direct or indirect) was obtained in a real food system; studies reporting only final antimicrobial or antioxidant efficacy are excluded from this column and are discussed in Section 5.

**Table 4:** Overview of primary delivery and application strategies for phenolic acids in food preservation.

Application Strategy	Process Overview	Preservation Outcomes	References
Direct incorporation	Phenolic acids are directly mixed into liquid or semi-solid foods via mechanical stirring.	Enhanced oxidative stability and suppression of lipid peroxidation; Inhibition of aerobic spoilage microbiota; Maintenance of color, texture, and physicochemical integrity; Potential replacement or reduction in synthetic curing agents without compromising sensory acceptance.	[75]
Encapsulation systems	Phenolic acids are entrapped within biopolymer-based carriers (e.g., polysaccharides, proteins, lipid nanoparticles) via spray drying, ionic gelation, nanoemulsion, or liposomal techniques.	Improved phenolic stability and protection against premature degradation; Sustained release behavior under storage conditions; Reduction in oxidative indicators; Suppression of spoilage and pathogenic microorganisms; Preservation of sensory and structural quality.	[76]
Edible coating incorporation	Phenolics are incorporated into film-forming matrices such as chitosan, gelatin, starch, or composite biopolymers, applied as coatings or standalone packaging films.	Significant inhibition of Gram-positive and Gram-negative bacteria; Reduced water loss, lipid oxidation, and protein degradation; Extended shelf life of fresh meat and fruits.	[77]
Active packaging films	Immobilization or controlled loading of phenolics into polymeric packaging materials, enabling gradual migration into food microenvironment.	Stimuli-responsive or competitive-release antimicrobial action; Suppression of biogenic amine accumulation and spoilage metabolite formation; Enhanced barrier performance against oxygen and ultraviolet; Improved inhibition of Gram-positive and Gram-negative bacteria including *Escherichia coli* and *Staphylococcus aureus*; Prolonged product stability under cold storage.	[78]

**Table 5:** Comparative performance of phenolic acids in different food matrices: Linking deterioration pathways to preservative mechanisms.

Food System	Primary Deterioration Pathways	Application Strategy	Function-Oriented Preservation Outcomes	References
Fresh fruits and vegetables	Enzymatic browning and nutrient loss; Microbial contamination; Physiological senescence	Coating/Immersion; Active packaging	Antioxidant activity: enhances the activities of superoxide dismutase; Activates host defense enzymes; Delays browning and nutrient loss. Antimicrobial activity: Disrupts fungal membrane integrity; Inhibits mycelial growth.	[86]
Fresh meat and poultry products	Lipid oxidation; Protein oxidation and degradation; Microbial spoilage	Coating/Immersion; Active packaging	Antioxidant activity: Breaks free radical chain reactions; Chelates pro-oxidant metal ions; Reduces thiobarbituric acid reactive substances and protein carbonyls. Antimicrobial activity: Suppresses total viable count and pathogens.	[40]
Aquatic products	Protein oxidation and degradation; Microbial imbalance; Lipid oxidation	Coating/Immersion; Active packaging	Antioxidant activity: Protects myofibrillar proteins by maintaining sulfhydryl groups from oxidation; Inhibits lipid oxidation. Antimicrobial activity: Suppresses specific spoilage organisms; Modulates microbial community dynamics.	[87,88]
Lipid-rich and emulsified foods	Lipid autoxidation; Interfacial oxidative instability; Flavor and nutrient loss	Direct addition; Encapsulation	Antioxidant activity: Provides effective radical scavenging; Delays hydroperoxide formation; Inhibits oxidative rancidity and maintains the physical stability of emulsion systems.	[89]

## Data Availability

No data was used for the research described in the article.

## Supplementary Materials

The following supporting information can be downloaded at https://www.mdpi.com/article/10.3390/foods15122100/s1, Table S1: Chemical information, dietary sources, and documented bioactivities of major PAs and phenolamides.
