# Supplementary material for: Dietary Plant-Derived Phenolic Acids and Phenolamides as Natural Preservatives: Antibacterial, Antioxidant and Food Preservation Applications
Source: Foods. 2026 Jun 11;15(12):2100. doi: 10.3390/foods15122100 (PMC13298898; doi:10.3390/foods15122100)
Supplement: Supplementary file 1 [file foods-15-02100-s001.zip › foods-4336680-supplementary.pdf]

Table S1. Chemical information, dietary sources, and documented bioactivities of major PAs and phenolamides

	No.	Compound name	Chemical structure	CAS No.	Dietary sources	Biological activities	References
Hydroxybenzoic acids	1	Salicylic acid	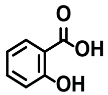	69-72-7	Fruits, Vegetables, Herbs, Spices	Antibacterial, Antifungal, Anti-inflammatory, Anticancer, Anti-proliferative, Antiviral, Analgesic	[12]
	2	Protocatechuic acid	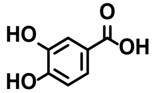	99-50-3	Fruits, Vegetables	Antibacterial, Anti-inflammatory, Antimutagenic, Antidiabetic, Antiulcer, Antiviral, Antifibrogenic, Antiallergic, neuroprotective, Anticancer, Anti-osteoporotic, Anti-aging, Analgesic	[13]
	3	Gallic acid	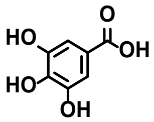	149-91-7	White tea, Black tea, Mango, Banana, Berries, Clove, Thyme, Chestnut	Antibacterial, Antioxidant, Antifungal, Anticancer, Cardioprotective, Neuroprotective, Anti-inflammatory, Antiviral	[14]
	4	Ellagic acid	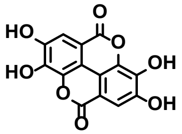	476-66-4	Pomegranates, Strawberries, Blackberries, Raspberries, Blueberries, Nuts, Seeds, Green tea	Antibacterial, Antioxidant, Antiestrogenic, Anti-proliferative, Anti-obesity, Anti-mutation, Antiallergic, Hepatoprotective, Cardioprotective, Chemopreventive, Gastroprotective,	[15]

						Antidiabetic, Neuroprotective	
Hydroxycinnamic acids	1	*p*-Coumaric acid	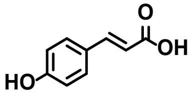	501-98-4	Tomatoes, Carrots, Cereals	Antibacterial, Antioxidant, Antifungal, Anticancer, Anti-inflammatory, Anti-aging skin, Anti-proliferative, Nephroprotective, Neuroprotective	[16]
	2	Caffeic acid	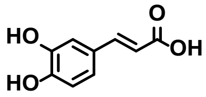	331-39-5	Coffee, Mint, Oregano, Rosemary, Thyme, Coriander, Cardamom, Blueberry, Yerba mate, Mango, Banana	Antibacterial, Antioxidant, Antifungal, Anti-inflammatory, Antihyperglycemic, Anticancer, Cardioprotective, Cytotoxic, Anticoagulant, Antidiabetic, Antiviral	[3]
	3	Ferulic acid	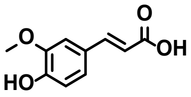	1135-24-6	Grasses, Grains, Vegetables, Flowers, Fruits, Leaves, Beans, Coffee bean, Artichoke, Peanut, Nuts	Antibacterial, Antioxidant, Antifungal, Anti-inflammatory, Anticancer, Antidiabetic, Cardioprotective, Neuroprotective	[17]
	4	Sinapinic acid	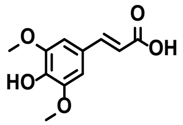	530-59-6	Rye, Fruits, Vegetables	Antibacterial, Antioxidant, Antifungal, Anticancer, Anti-inflammatory, Neuroprotective, Anxiolytic, Cardioprotective	[18]

	5	Chlorogenic acid	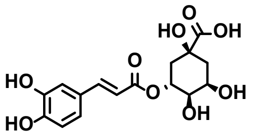	327-97-9	Apple, Peach, Pineapple, Blueberry, Coffee, Sunflower	Antibacterial, Antioxidant, Anti-inflammatory, Anti-obesity, Anticancer, Cardioprotective, Hepatoprotective, Metabolic regulatory, Antidiabetic, Neuroprotective	[19]
	6	Rosmarinic acid	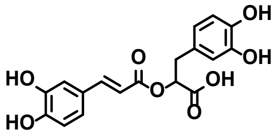	20283-92-5	*Malva sylvestris*, *Melissa officinalis*, *Salvia officinalis*, *Rosmarinus officinalis*, *Coleus blumei*	Antibacterial, Antioxidant, Antifungal, Sedative, Hypnotic, Antiepileptic, Antiviral, Analgesic, Antimutagenic, Antiallergic, Anti-inflammatory, Anti-tumor, Anti-Parkinson, Antiangiogenic, Neuroprotective, Antidepressant, Cognitive-enhancing, Metabolic-regulating	[20]
Phenolamides	1	Avenanthramide A	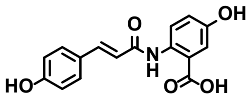	108605-70-5	Oat	Antioxidant, Anti-atherogenic, Anti-proliferative, Anti-inflammatory	[21]
	2	Avenanthramide C	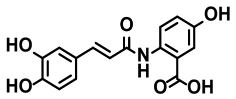	116764-15-9	Oat	Antioxidant, Anti-inflammatory, Anti-atherogenic, anti-apoptotic, neuroprotective properties, Anticancer	[22]

3	*N-p-trans*-Coumaroyltyramine	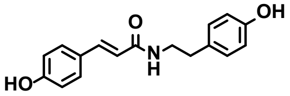	36417-86-4	*Solani melongenae* radix, *Corydalis edulis*, *Peperomia tetraphylla*, *Annona cherimola*, *Tribulus terrestris*, *Annona montana*, Chinese yam, Garlic, Welsh onion	Antibacterial, Antioxidant, Anti-inflammatory, Anticancer	[23]
4	*N*-Caffeoyltyramine	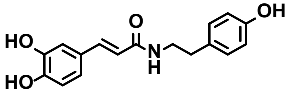	103188-48-3	*Annona crassiflora* seeds, *Annona montana*, *Annona cherimola*, *Lycium chinense*, *Vitis trifolia*, Hemp	Antibacterial, Antioxidant, Anti-melanogenic, Anti-inflammatory, Anti-aging, Dermato-protective	[24]
5	*N-p-trans*-Coumaroyloctopamine	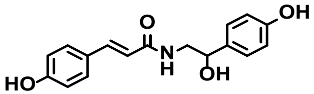	66648-45-1	*Lycianthes biflora*, *Phellodendron chinense*, *Celtis occidentalis*, *Lycium chinense*, *Polygonatum odoratum*, Eggplant, Garlic skin	Antioxidant	[25]
6	*N-trans*-Caffeoyloctopamine	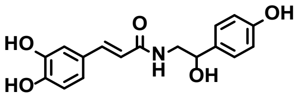	1378868-10-0	*Solanum melongena* L.	Antioxidant	[26]
7	*N-trans*-Caffeoyldopamine	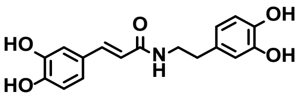	103188-49-4	*Capsicum annuum*, *Theobroma cacao*, *Lycium chinense*	Antibacterial, Antioxidant, Antifungal, Anti-inflammatory, Neuroprotective, Anticancer	[27]

8	*N-trans*-Feruloyltyramine	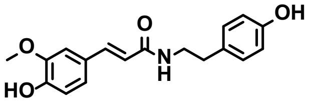	65646-26-6	*Balanites aegyptiaca*, *Hibiscus tiliaceus*, *Litsea greenmaniana*, *Polygonum hyrcanicum*, *Corydalis edulis*, *Cornulaca monacantha*, Potato, Sugar beet	Antioxidant, Antibacterial, Antifungal, Anticancer, Neuroprotective, Cholinergic-promoting, Anti-inflammatory	[28]
9	*N-trans*-Feruloyloctopamine	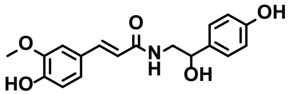	66648-44-0	*Melochia umbellata*, *Portulaca oleracea* L., *Polygonatum odoratum*, *Celtis occidentalis* L., Garlic skin, Potato	Antioxidant, Antidiabetic, Anticancer	[29]
10	*N-trans*-Feruloyldopamine	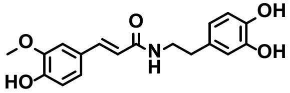	142350-99-0	*Corydalis impatiens*, *Arundo donax* L., *Atraphaxis spinosa* L.	Antioxidant	[30]
11	*N*-Coumaroylserotonin	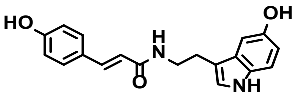	68573-24-0	*Centaurea*, Japanese barnyard millet, Safflower, Konnyaku,	Antioxidant	[31]
12	*N-trans*-Sinapoyltyramine	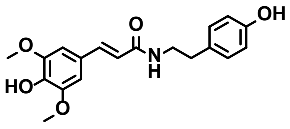	200125-11-7	*Peperomia tetraphylla*, *Porcelia macrocarpa*, *Tetragonia tetragonioides*, *Corydalis edulis*, *Corydalis impatiens*, *Lindera glauca*, *Amaranthus spp.*,	Antibacterial, Anticancer	[30]

13	Di-feruloylputrescine	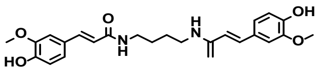	42369-86-8	*Haloxylon articulatum*, Shallot Corn bran, Mousouchiku	Anticancer, antioxidant	[32]
14	Lyciumamide A	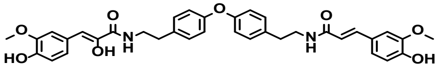	1647111-40-7	*Lycium barbarum*	Antioxidant	[33]
